# Sexual dimorphisms in serum calcium and phosphate concentrations in the Rotterdam Study

**DOI:** 10.1038/s41598-023-34800-w

**Published:** 2023-05-23

**Authors:** Ariadne Bosman, W. Nadia H. Koek, Natalia Campos-Obando, Bram C. J. van der Eerden, M. A. Ikram, André G. Uitterlinden, Johannes P. T. M. van Leeuwen, M. C. Zillikens

**Affiliations:** 1 Department of Internal Medicine, Erasmus MC, University Medical Center Rotterdam, PO Box 2040, 3000 CA Rotterdam, The Netherlands; 2 Department of Epidemiology, Erasmus MC, University Medical Center Rotterdam, Rotterdam, The Netherlands

**Keywords:** Endocrine system and metabolic diseases, Calcium and vitamin D

## Abstract

Sex differences in serum phosphate and calcium have been reported but the exact nature and underlying regulatory mechanisms remain unclear. We aimed to compare calcium and phosphate concentrations between sexes, and explore potential covariates to elucidate underlying mechanisms of sex differences in a prospective, population-based cohort study. Pooled data of subjects > 45 years from three independent cohorts of the Rotterdam Study (RS) were used: RS-I-3 (n = 3623), RS-II-1 (n = 2394), RS-III-1 (n = 3241), with separate analyses from an additional time point of the first cohort RS-I-1 (n = 2688). Compared to men, women had significantly higher total serum calcium and phosphate concentrations which was not explained by BMI, kidney function nor smoking. Adjustment for serum estradiol diminished sex differences in serum calcium while adjustment for serum testosterone diminished sex differences in serum phosphate. Adjustment for vitamin D and alkaline phosphatase did not change the association between sex and calcium or phosphate in RS-I-1. In the sex-combined group, both serum calcium and phosphate decreased with age with a significant interaction for sex differences for serum calcium but not phosphate. In sex-stratified analyses, serum estradiol but not testosterone was inversely associated with serum calcium in both sexes. Serum estradiol was inversely associated with serum phosphate in both sexes to a similar degree, while serum testosterone was inversely associated with serum phosphate in both sexes with an apparent stronger effect in men than in women. Premenopausal women had lower serum phosphate compared to postmenopausal women. Serum testosterone was inversely associated with serum phosphate in postmenopausal women only. In conclusion, women > 45 years have higher serum calcium and phosphate concentrations compared to men of similar age, not explained by vitamin D or alkaline phosphatase concentrations. Serum estradiol but not testosterone was inversely associated with serum calcium while serum testosterone was inversely associated with serum phosphate in both sexes. Serum testosterone may in part explain sex differences in serum phosphate while estradiol could partly explain sex differences in serum calcium.

## Introduction

Calcium and phosphate are important electrolytes in human physiology. Calcium is one of the most abundant cations in the body. It is crucial for several metabolic processes such as neural transmission, blood coagulation and cell proliferation, and pivotal for bone mineralization. Approximately 51% of total calcium is bound to proteins such as albumin and globulin while the remainder circulates in ionic form (free serum calcium or Ca^2+^)^1^. Serum calcium concentrations are tightly controlled through the interaction of the intestines, kidneys, bone, and parathyroid glands. Parathyroid hormone (PTH) and 1,25-dihydroxyvitamin D (1,25(OH)_2_D_3_) are the major hormones controlling calcium homeostasis by stimulating calcium absorption in the intestines, calcium reabsorption in the kidney and bone resorption.

Phosphate is an important electrolyte in energy metabolism and is part of DNA and RNA structures^2,3^. Moreover, it is incorporated in extracellular matrix as hydroxyapatite during bone formation. Serum calcium and phosphate concentrations influence each other^1,2^. Serum phosphate is predominantly controlled through urinary excretion by the actions of PTH and the osteocyte-derived hormone fibroblast growth factor 23 (FGF23). Additionally, 1,25(OH)_2_D_3_ regulates phosphate absorption from the diet in the intestine^2^. Phosphate is present in serum mainly in a free and ultra-filterable form (85–90%), whilst the remainder (10–15%) is bound to proteins^3,4^.

Serum calcium and phosphate imbalance has been linked to several disorders such as cardiovascular disease, COPD, metabolic syndrome and osteoporosis, and mortality^4–9^. Furthermore, the calcium-phosphate product is associated with morbidity and mortality in patients with end-stage renal disease^7^, although the recent KDIGO guideline suggests no additional value of this construct over individual serum concentrations of calcium and phosphate in patients with chronic kidney disease (CKD)^10^. Some of these disorders display marked sex-specific incidences: the incidence of cardiovascular diseases has been found to be lower in women compared to men^5,11^. Moreover, Tohno et al. found sex differences in the accumulation of calcium and phosphate in coronary arteries^12^. Thus, it is possible that sex differences in the incidence of these disorders might be related to calcium and phosphate homeostasis and that the underlying mechanisms may have clinical consequences.

There have been several studies evaluating sex differences in serum calcium and phosphate. Although these studies found sex differences between post-menopausal women and men, the findings were less consistent for serum calcium than for serum phosphate^4,13–15^. Studies evaluating total serum calcium concentrations have found differences between pre- and postmenopausal women, and between men and women at various ages, but these results were either inconsistent or sex differences were not systematically investigated in different age groups: Lindgarde et al. and Roof et al. reported that serum total calcium was significantly higher in young men compared to women, while Håglin et al. found that women had higher serum calcium concentrations compared to men^13,16–19^. More consistent data are available showing that postmenopausal women have higher serum phosphate concentrations compared to men of similar age^5,18,20–23^. In a previous study conducted in three different samples extracted from a hospital information system, we found that, above 45 years of age, women have higher calcium and phosphate concentrations than men^24^.

In this study, we compared serum calcium and phosphate concentrations between men and women in a population-based cohort study of elderly white Caucasians, i.e., the Rotterdam Study, and explored the role of potential confounders including age, vitamin D and sex hormones and how they influence calcium and phosphate concentrations in both sexes.

## Methods

### Rotterdam study

The Rotterdam Study (RS) is a large prospective population-based cohort study of Caucasian subjects aged 45 years and older, living in the Ommoord district of Rotterdam, The Netherlands. The study was designed to investigate the incidence and determinants of chronic disabling diseases in the elderly. Rationale and design have been described previously^25^. It is now composed of four cohorts. The first cohort, named RS-I, initiated in 1989. RS-II, RS-III and RS-IV followed in 2000, 2005 and 2017, respectively. Participants have been followed through several visits since recruitment, which are all similar in design and data collection.

Serum phosphate and calcium concentrations have been measured in the baseline visit of RS-I, named RS-I-1; in the second follow up visit of RS-I, named RS-I-3; and in the baseline visits of RS-II and RS-III. The participants in RS-I and RS-II are all aged 55 years and older, while RS-III consists of participants aged 45 years and older. Serum creatinine, 25-hydroxyvitamin D (25(OH)D) and estradiol and testosterone concentrations were measured in all cohorts. All measurements were taken in a fasting state, except for RS-I-1. A total of 3623 participants from RS-I-3, 2394 from RS-II and 3241 from RS-III with complete information on fasting serum calcium, serum phosphate and covariates were included in the main analyses of this study.

A total of 2688 participants from RS-I-1 had information on non-fasting serum calcium, phosphate and covariates. In addition, serum albumin, alkaline phosphatase (ALP) and 1,25(OH)_2_D_3_ concentrations were measured only in RS-I-1.

### Assay methods

Serum samples from subjects were analyzed directly after sample collection at the Department of Internal Medicine of the Erasmus MC Rotterdam, The Netherlands.

Serum total calcium, inorganic phosphate, albumin and ALP were measured using the Hitachi 917 Analyzer (Roche, Mannheim, Germany). The corresponding inter-assay coefficients of variation (CV) were < 0.7% for serum calcium and phosphate. The calcium-phosphate product was calculated by multiplying the subjects’ total serum calcium and phosphate concentrations, and expressed in mmol^2^/L^2^. Since Payne reported in 1973 that serum albumin influences serum calcium concentrations, we calculated corrected serum calcium concentrations using the formula: calcium (corrected) = calcium measured + 0.02 (42—albumin measured) in RS-I-1^26^.

In RS-I-3, RS-II-1 and RS-III-1, serum 25(OH)D concentrations were measured using electrochemiluminescence immunoassays (COBAS, Roche Diagnostics GmbH, Germany). The inter-assay CV for 25(OH)D was < 7.8%. For RS-I-1, serum 1,25(OH)_2_D_3_ and 25(OH)D concentrations were measured using 125I-radioimmunoassays (RIA) with inter-assay CVs of 7–11% and 7.9% (IDS, Boldon, UK and DiaSorin, Stillwater, MN, USA; respectively)^27,28^.

Total estradiol concentrations were determined using a COBAS 8000 Modular Analyzer (Roche Diagnostics GmbH) with a lower limit of detection of 18.4 pmol/L. Total testosterone concentrations were determined using liquid chromatography-tandem mass spectrometry (LC–MS/MS). The inter-assay CVs were < 4.6% for serum estradiol and < 5.7% for serum testosterone^29^.

Due to limited amount of plasma per subject in RS-I-1, not all hormone concentrations could be determined in all subjects.

### Assessment of covariates

BMI was calculated as the body mass divided by the square of the body height, measured in standing position without shoes, and expressed in kg/m^2^. eGFR (mL/min/1.73 m^2^) was estimated using the Chronic Kidney Disease Epidemiology Collaboration equations based on creatinine concentrations^30,31^. In all cohorts, smoking status was assessed during home interviews. Women were interviewed for having previously used hormone replacement therapy (HRT). No data on current use of HRT was documented. In RS-II-1 and RS-III-1, women were interviewed for their current menopausal status. Women were considered perimenopausal if they were within one year of their last menstruation.

### Statistical analyses

Data from RS-I-3, RS-II-1 and RS-III-1 were pooled (and from now on called “the pooled dataset”) in order to evaluate overall sex differences for serum calcium and phosphate. RS-I-I was analyzed separately because of non-fasting condition and availability of additional covariates. Differences between men and women were tested with independent t-tests for continuous variables and chi-square tests for categorical variables. Multivariate linear regression models were used to study sex differences in serum calcium and phosphate. All analyses were performed using Z-scores for the biomarkers, calculated by subtracting the mean from the value divided by the standard deviation. The analyses included covariates that are known to differ between the sexes and covariates that potentially influence the outcome variables serum calcium and phosphate: age, BMI, smoking and eGFR. Analyses were additionally adjusted for cohort to correct for unexpected categorical effects, with RS-I-3 being given dummy variables coded 0–0–1, RS-II-1 given dummy variables coded 0–1–0, and RS-III-1 given dummy variable 1–0–0. Analyses were performed in the total population and sex-stratified. In the total population, we included interaction terms of age with sex to explore whether sex differences in serum calcium and phosphate vary according to age. In order to evaluate the influence of sex hormones and 25(OH)D on sex differences in serum calcium and phosphate concentrations, we tested the change in the beta-coefficient of observed sex differences in a multivariate linear regression model in the total population. A change of > 20% in the beta-coefficient of sex, after correction for the specific variable, indicated that the variable has an important influence on the observed sex differences. Because sex hormone concentrations are associated with sex we tested for multicollinearity. A variance inflation factor (VIF) of 10 was applied as a threshold for multicollinearity^32^.

To evaluate age in relation with serum calcium and phosphate, subjects in the pooled dataset were stratified into consecutive decades and stratified by sex. Age-group differences were tested per sex using ANCOVA in a general linear model and adjusted for BMI, smoking, eGFR and cohort.

Sex stratified two-tailed partial correlation analyses in the pooled dataset were performed to test for correlations between serum 25(OH)D, estradiol and testosterone and serum calcium and phosphate. In order to study the influence of sex hormones and 25(OH)D on serum calcium and phosphate in men and women separately, we performed sex-stratified multivariate regression analyses adjusting for serum estradiol, testosterone and 25(OH)D. Analyses in women were additionally adjusted for previous hormone replacement therapy (HRT) and menopausal status. Furthermore, analyses were performed in pre- and postmenopausal women separately. Lastly, we compared beta-coefficients for age and serum calcium or phosphate with and without addition of serum estradiol, testosterone and 25(OH)D. All results are expressed per 1-SD increase of serum estradiol, testosterone or 25(OH)D, unless otherwise stated.

### Sensitivity analyses

All analyses were also performed in RS-I-1 and were further adjusted for serum 25(OH)D, 1,25(OH)_2_D_3_ and ALP. Data on sex hormones as well as 25(OH)D and 1,25(OH)_2_D_3_ were only available in a subset of RS-I-1 (Supplementary Table 8). Because a large proportion of serum calcium is bound to albumin, sex differences in serum albumin concentrations were analyzed and serum calcium concentrations were corrected for serum albumin in RS-I-1. Furthermore, analyses were repeated in the subset of RS-I-1 with albumin concentrations within a narrow range (38–42 g/L).

Statistical analyses were performed using SPSS (version 23). Due to the explorative nature of the study, statistical significance of two-sided tests was defined as *P < *0.05.

### Ethical approval

The Rotterdam Study has been approved by the Medical Ethics Committee of the Erasmus MC (registration number MEC 02.1015) and by the Dutch Ministry of Health, Welfare and Sport (Population Screening Act WBO, license number 1071272-159521-PG). The Rotterdam Study has been entered into the Netherlands National Trial Register (NTR; www.trialregister.nl) and into the WHO International Clinical Trials Registry Platform (ICTRP; https://apps.who.int/trialsearch/) under shared catalogue number NTR6831. All participants provided written informed consent to participate in the study and to have their information obtained from treating physicians. All methods were carried out in accordance with relevant guidelines and regulations.

## Results

The general characteristics of the pooled dataset are described in Table 1. The general characteristics of the individual cohorts are described in Supplemental Tables 1–3. Women had higher serum calcium and phosphate concentrations compared to men (mean (SD) serum calcium: 2.42 (0.10) mmol/L in men, 2.44 (0.10) mmol/L in women, *P < *0.001; mean(SD) serum phosphate: 1.02 (0.15) mmol/L in men, 1.17(0.15) mmol/L in women, *P < *0.001). Consequently, the calcium-phosphate product was higher in women compared to men. Serum 25(OH)D, estradiol and testosterone concentrations were significantly lower in women compared to men. The majority of women was postmenopausal.

**Table 1:** Baseline characteristics of the pooled dataset.

	Men	Women	*P-value*
N	4043	5215	
Age (years)	64.5 (9.4)	65.3 (9.8)	< 0.001
Calcium (mmol/L)	2.42 (0.10)	2.44 (0.10)	< 0.001
Phosphate (mmol/L)	1.02 (0.15)	1.17 (0.15)	< 0.001
Calcium-phosphate product (mmol^2^/L^2^)	2.47 (0.39)	2.86 (0.39)	< 0.001
25(OH)D (nmol/L)	59.9 (27.9)	53.5 (27.2)	< 0.001
eGFR (mL/min/1.73 m^2^)	80.0 (16.2)	78.7 (15.9)	< 0.001
Estradiol (pmol/L)	104.2 (40.7)	64.5 (102.5)	< 0.001
Testosterone (nmol/L)	17.17 (5.96)	0.94 (0.81)	< 0.001
BMI (kg/m^2^)	27.0 (3.6)	27.5 (4.6)	< 0.001
Current smoking, n(%)	805 (19.9%)	992 (19.0%)	0.384
Ever HRT use, n(%)		847 (18.2%)	
Postmenopausal, n(%)	–	4802 (92.1%)	

Continuous values are displayed as mean (standard deviation)), categorical variables are displayed in absolute counts (%). Differences between males and females were analyzed using an independent t test for continuous variables and χ2 test for categorical variables. Abbreviations: 25(OH)D, 25-hydroxyvitamin D; 1,25(OH)_2_D_3_, 1,25-dihydroxyvitamin D; BMI, body mass index; eGFR, estimated glomerular filtration rate; HRT, hormone replacement therapy; ND, not determined; RS, Rotterdam Study.

### Sex differences in serum calcium and serum phosphate

In multivariate linear regression analyses in the total population, we found a significant association of sex on serum calcium and phosphate (serum calcium: β 0.274, 95%CI 0.234 to 0.314; *P < *0.001; serum phosphate: β 0.917, 95%CI 0.881 to 0.953; *P < *0.001). Moreover, age was inversely associated with both serum calcium and serum phosphate concentrations (serum calcium: β − 0.120, 95%CI − 0.152 to − 0.088; *P < *0.001; serum phosphate: β − 0.038, 95%CI − 0.067 to − 0.009; *P < *0.001) (Table 2). Interaction analysis of age and sex showed an interaction for age and sex on serum calcium concentrations with a standardized beta coefficient of 0.43 *(P < *0.001) for the interaction term age*sex. For analyses with serum phosphate modelled as the outcome, the interaction term age*sex was not significant *(P* = 0.3). Next, we analyzed the influence of serum testosterone, estradiol and 25(OH)D concentrations on sex differences in serum calcium and phosphate concentrations in the total population (Table 3). Multicollinearity statistics showed a VIF of 1.068 for serum estradiol and 5.077 for serum testosterone. Serum estradiol diminished the effect size for the association between sex and serum calcium by 20.4% (β 0.218, 95%CI 0.177 to 0.259; *P < *0.001), while serum testosterone enhanced the effect size by 32.8%. The effect size for the association between sex and serum phosphate did not change after adjusting for serum estradiol. Adjusting for serum testosterone diminished the effect size of the association between sex and serum phosphate by 37.6% but it remained significant (β 0.559, 95%CI 0.478 to 0.640; *P < *0.001). Serum 25(OH)D did not change the effect size for the associations between either sex and serum calcium or sex and serum phosphate.

**Table 2:** Association between sex and age, and serum calcium and phosphate concentrations in the pooled dataset.

	Serum calcium (mmol/L)		Serum phosphate (mmol/L)	
Pooled dataset N = 9258				
	Standardized Beta-coefficient (95% CI)	*P* value	Standardized Beta-coefficient (95% CI)	*P* value
Sex	0.274 (0.234 to 0.314)	< 0.001	0.917 (0.881 to 0.953)	< 0.001
Age	− 0.120 (− 0.152 to − 0.088)	< 0.001	− 0.038 (− 0.067 to − 0.009)	0.011

βs were obtained from linear regression models. All models were adjusted for sex, age in decades, cohort, BMI, eGFR and smoking.

**Table 3:** Influence of serum testosterone, estradiol and 25(OH)D on sex differences in serum calcium and phosphate concentrations in the pooled dataset.

	Serum calcium (mmol/l)		Serum phosphate (mmol/l)	
Pooled dataset N = 9270				
	Standardized Beta-coefficient sex (95% CI)	% change beta-coefficient from model 1 (%)	Standardized Beta-coefficient sex (95% CI)	% change beta-coefficient from model 1 (%)
Model 1^a^	0.274 (0.234 to 0.314)		0.917 (0.881 to 0.953)	
Model 2^b^	0.218 (0.177 to 0.259)	− 20.4	0.856 (0.819 to 0.893)	− 6.7
Model 3^c^	0.364 (0.274 to 0.455)	32.8	0.572 (0.490 to 0.654)	− 37.6
Model 4^d^	0.351 (0.261 to 0.441)	28.1	0.559 (0.478 to 0.640)	− 39.0
Model 5^e^	0.284 (0.244 to 0.3240	3.6	0.915 (0.879 to 0.951)	− 0.2

βs were obtained from linear regression models and expressed per 1-SD increase in serum estradiol, testosterone or 25(OH)D. ^a^Model 1: adjusted for age in decades, cohort, BMI, eGFR and smoking. ^b^Model 2: model 1 and estradiol. ^c^Model 3: model 1 and testosterone. ^d^Model 4: model 1 and estradiol and testosterone. ^e^Model 5: model 1 and 25(OH)D.

After stratification of subjects into age in decades, serum calcium and phosphate showed a relation with age in both men and women, with calcium and phosphate concentrations being lower in each consecutive decade (Supplementary Table 4).

In sex-stratified correlation analyses, a negative correlation was found between serum estradiol and serum calcium in both sexes (men: *ρ* − 0.110, *P < *0.001; women: *ρ* − 0.121, *P < *0.001), but not between testosterone and serum calcium. Serum 25(OH)D concentrations were positively correlated with serum calcium in both sexes (men: *ρ* 0.040, *P* = 0.01; women: *ρ* 0.070, *P < *0.001). Both serum estradiol and testosterone were negatively correlated with serum phosphate in both sexes (for serum estradiol: men: *ρ* − 0.122, *P < *0.001; women: *ρ* − 0.142, *P < *0.001; for serum testosterone: men: *ρ* − 0.118, *P < *0.001; women: *ρ* − 0.046, *P < *0.001) (Table 4). Serum 25(OH)D concentrations were negatively correlated with serum phosphate in men only. In correlation analysis stratified by menopausal status, serum estradiol was negatively correlated with serum calcium and serum phosphate in both pre- and postmenopausal women. Serum testosterone was not correlated with serum calcium in both pre- and postmenopausal women, but it was negatively correlated with serum phosphate in postmenopausal women (*ρ* − 0.049, *P < *0.001). Serum 25(OH)D concentrations were positively correlated with serum calcium but not with serum phosphate in both pre- and postmenopausal women.

**Table 4:** Partial correlation analysis in the pooled data set of RS-I-3, RS-II-1 and RS-III-1 for serum calcium and phosphate with 25(OH)D, estradiol and testosterone, stratified by sex and menopausal status.

	Serum calcium		Serum phosphate	
	R	*P* value*	R	*P* value*
By sex				
Men				
Estradiol	− 0.110	< 0.001	− 0.122	< 0.001
Testosterone	0.028	0.080	− 0.118	< 0.001
25(OH)D	0.040	0.010	− 0.039	0.014
Women				
Estradiol	− 0.121	< 0.001	− 0.142	< 0.001
Testosterone	0.004	0.785	− 0.046	< 0.001
25(OH)D	0.070	< 0.001	0.016	0.248
By menopausal status				
Premenopausal women				
Estradiol	− 0.109	0.045	− 0.127	0.019
Testosterone	0.046	0.397	0.052	0.338
25(OH)D	0.080	0.138	0.011	0.845
Postmenopausal women				
Estradiol	− 0.136	< 0.001	− 0.155	< 0.001
Testosterone	0.003	0.858	− 0.049	< 0.001
25(OH)D	0.067	< 0.001	0.015	0.300

*Analyses are adjusted for age, BMI, eGFR, smoking and cohort (and menopausal status and HRT use in women).

Sex-stratified multivariate linear regression analyses revealed an inverse association of age on serum calcium in both sexes, while age was inversely associated with serum phosphate in women only (Table 5). Adding serum testosterone to the model revealed that serum testosterone was not associated with serum calcium in both sexes, but it was inversely associated with serum phosphate in both sexes (men: β − 0.123, 95%CI − 0.155 to − 0.091; *P < *0.001; women: β − 0.047, 95%CI − 0.073 to − 0.021; *P < *0.001). Serum estradiol was inversely associated with serum calcium and serum phosphate in both sexes. Serum 25(OH)D was positively associated with serum calcium in both sexes and negatively associated with serum phosphate in men only. Serum testosterone, estradiol and 25(OH)D did not influence the effect size for age on serum calcium in men. (Supplementary Table 5).

**Table 5:** Sex-stratified beta-coefficients for serum calcium and phosphate concentrations, according to covariates, in the pooled dataset.

	Serum calcium (mmol/L)		Serum phosphate (mmol/L)	
Men (n = 4043)				
	Standardized Beta-coefficient (95% CI)	*P* value	Standardized Beta-coefficient (95% CI)	*P* value
Age (per decade)	− 0.166 (− 0.217 to − 0.116)	< 0.001	− 0.047 (− 0.098 to 0.005)	0.075
Testosterone	0.028 (− 0.003 to − 0.060)	0.081	− 0.123 (− 0.155 to − 0.091)	< 0.001
Estradiol	− 0.113 (− 0.146 to − 0.081)	< 0.001	− 0.132 (− 0.165 to − 0.098)	< 0.001
25(OH)D	0.040 (0.009 to 0.070)	0.011	− 0.039 (− 0.070 to − 0.008)	0.013
Women (n = 5215)				
	Standardized Beta-coefficient (95% CI)	*P* value	Standardized Beta-coefficient (95% CI)	*P* value
Age (per decade)	− 0.119 (− 0.163 to − 0.075)	< 0.001	− 0.081 (− 0.124 to − 0.038)	< 0.001
Testosterone	0.004 (− 0.023 to 0.031)	0.774	− 0.047 (− 0.073 to − 0.021)	< 0.001
Estradiol	− 0.104 (− 0.134 to − 0.073)	< 0.001	− 0.131 (− 0.160 to − 0.101)	< 0.001
25(OH)D	0.077 (0.048 to 0.107)	< 0.001	0.014 (− 0.015 to 0.043)	0.344

βs were obtained from linear regression models and expressed per 1-SD increase in serum estradiol, testosterone or 25(OH)D. All models were adjusted for age, cohort, BMI, eGFR and smoking (and menopausal status and previous HRT use in women).

Analyses in women were further stratified by menopausal status (Table 6, Supplementary Table 6). Postmenopausal women had higher serum phosphate and testosterone concentrations and higher calcium-phosphate product levels than premenopausal women. Serum 25(OH)D and estradiol was lower in postmenopausal women. Age was positively associated with serum calcium in premenopausal women and inversely associated with serum calcium and serum phosphate in postmenopausal women. Serum testosterone was not significantly associated with serum calcium nor serum phosphate in premenopausal women but there was a significant inverse association between serum testosterone and phosphate in postmenopausal women (β − 0.048, 95%CI − 0.075 to − 0.020; *P < *0.001). Estradiol was inversely associated with serum calcium and phosphate in postmenopausal women but in premenopausal women only with serum phosphate and not serum calcium. Serum 25(OH)D was positively associated with serum calcium in both pre- and postmenopausal women but not with serum phosphate.

**Table 6:** Beta-coefficients, stratified by menopausal status, for serum calcium and phosphate concentrations, according to covariates, in the pooled dataset.

	Serum calcium (mmol/L)		Serum phosphate (mmol/L)	
Premenopausal (n = 413)				
	Standardized Beta-coefficient (95% CI)	*P* value	Standardized Beta-coefficient (95% CI)	*P* value
Age (per decade)	0.360 (0.022 to 0.699)	0.037	0.308 (− 0.026 to 0.642)	0.070
Testosterone	0.038 (− 0.060 to 0.137)	0.448	− 0.027 (− 0.124 to 0.070)	0.587
Estradiol	− 0.072 (− 0.169 to 0.025)	0.143	− 0.099 (− 0.195 to − 0.004)	0.041
25(OH)D	0.102 (0.001 to 0.203)	0.048	− 0.013 (− 0.114 to 0.087)	0.793
Postmenopausal (n = 4802)				
	Unstandardized Beta-coefficient age (95% CI)	*P* value	Unstandardized Beta-coefficient age (95% CI)	*P* value
Age (per decade)	− 0.129 (− 0.173 to − 0.084)	< 0.001	− 0.087 (− 0.131 to − 0.043)	< 0.001
Testosterone	0.003 (− 0.025 to 0.031)	0.843	− 0.048 (− 0.075 to − 0.020)	< 0.001
Estradiol	− 0.128 (− 0.157 to − 0.099)	< 0.001	− 0.162 (− 0.190 to − 0.134)	< 0.001
25(OH)D	0.073 (0.042 to 0.104)	< 0.001	0.015 (− 0.015 to 0.046)	0.314

βs were obtained from linear regression models and expressed per 1-SD increase in serum estradiol, testosterone or 25(OH)D. All models were adjusted for age, cohort, BMI, eGFR and smoking (and previous HRT use in postmenopausal women).

### Sensitivity analyses

The baseline characteristics of RS-I-1 are depicted in Supplementary Table 7. Non-fasting serum calcium and phosphate concentrations were higher in women than in men (mean (SD) serum calcium: 2.36 (0.14) mmol/L in men, 2.37 (0.13) mmol/L in women, *P* = 0.002; mean (SD) serum phosphate: 1.09 (0.19) mmol/L in men, 1.23 (0.17) mmol/L in women, 0 < 0.001). Concentrations of 1,25(OH)_2_D_3_ were not significantly different between the sexes. ALP concentrations were higher in women compared to men (*P < *0.001). Serum 25(OH)D, testosterone and estradiol concentrations were lower in women than in men. Multivariate linear regression analysis showed a significant positive association of sex on serum calcium and serum phosphate. Age was negatively associated with serum phosphate. The association between age and serum calcium did not reach significance (Supplementary Table 8). The effect size of the association between sex and serum calcium and phosphate did not change after adjusting for serum 25(OH)D, serum 1.25(OH)_2_D_3,_ serum 25(OH)D and 1.25(OH)_2_D_3_, or ALP (Supplementary Table 9).

Lastly, we evaluated sex differences in serum albumin concentrations. Serum albumin concentrations were not significantly different between men and women. After correcting serum calcium for serum albumin concentrations according to the aforementioned formula, the sex differences in serum calcium concentrations remained, with women having higher corrected serum calcium concentrations compared to men (data not shown).

To exclude an impact of high or low albumin concentrations on calcium concentrations, we performed sensitivity analyses, restricting our data to only those subjects with albumin concentrations within a narrow range (38–42 g/L). In this subset, accounting for 42% of the total RS-I-1 dataset, we found similar results as in the overall data (mean (SD) serum calcium: 2.34 (0.13) mmol/L in men, 2.36 (0.13) mmol/L in women, *P* = 0.04).

## Discussion

In this study, sexual dimorphism in serum calcium and phosphate concentrations and the role of potential confounders were investigated. In a pooled analysis of subjects aged 45 years and older in three cohorts from the Rotterdam Study, we showed that serum calcium, phosphate and the calcium-phosphate product were significantly higher in women compared to men. Both serum calcium concentrations, in a sex-dependent manner, and serum phosphate concentrations, in a sex-independent manner, declined with aging. There are a few older and mainly small-sized studies that have found higher serum calcium concentrations in elderly women compared to men, although not always consistently^16,18,19^, while several studies have shown that postmenopausal women have higher serum phosphate concentrations than men of similar age^5,18,20–23,33^. Recently, we showed that serum calcium and phosphate are higher in women than in men in three samples extracted from a hospital information system^24^. The findings from the current study have now confirmed sexual dimorphism both in serum calcium and phosphate concentrations at the population level.

Sex hormone actions are a potential cause of the observed sex differences in serum calcium and phosphate concentrations. We found that serum estradiol diminished the sex differences in serum calcium while serum testosterone increased sex differences in serum calcium. In sex-stratified analyses we found that serum estradiol but not testosterone was inversely associated with serum calcium in both sexes. Sex differences in serum phosphate concentrations were not modified by serum estradiol concentrations but decreased after adjusting for serum testosterone concentrations. Sex-stratified analyses showed that serum estradiol was inversely associated with serum phosphate to a similar degree in both sexes, while the association between serum testosterone and serum phosphate appeared stronger in men than in women. Our findings are in line with a study of 59 elderly men with suppressed endogenous production of estradiol and testosterone through administration of a long-acting GnRH agonist. This study showed that depletion of estrogen but not testosterone in these men resulted in increased serum calcium concentrations and increased bone resorption markers, while both estradiol and testosterone suppression independently increased serum phosphate concentrations^34^. Thus, both estradiol and testosterone may play a role in the regulation of serum calcium and phosphate but to what extent and whether there are sex differences in the regulatory pathways remains unclear.

Calcium homeostasis is predominantly regulated at the level of the parathyroid glands, the kidneys, the intestines and bone. Both human and animal studies have found that estradiol reduces renal calcium excretion and increases intestinal calcium absorption suggesting sex differences in renal and intestinal calcium handling^35,36^. However, this is not in line with our findings of higher concentrations of serum calcium in postmenopausal women when estradiol concentrations drop, suggesting an additional regulatory mechanism. Nordin et al. postulated that the sexual dimorphism in calcium handling could be due to a change in PTH and increased sensitivity for the PTH action on bone after menopause^15,37^. Unfortunately, serum PTH concentrations were not available in our cohort. Interestingly, we observed that serum 25(OH)D was lower in women compared to men, while 1,25(OH)_2_D_3_ was not significantly different between sexes. This may indicate that there is increased conversion of 25(OH)D into 1,25(OH)_2_D_3_ in women compared to men, which could be mediated by PTH. Higher PTH concentrations in women could also explain the higher serum calcium concentrations. However, adjustments for both 25(OH)D and 1,25(OH)2D_3_ did not influence sex differences in serum calcium and phosphate.

Our data suggests that differences in sex hormones, especially serum testosterone, may partly explain the sex differences in serum phosphate above the age of 45. We cannot conclude with certainty that serum testosterone but not serum estradiol is important in the age-related sex differences of phosphate regulation since testosterone can be converted into estradiol through the activity of the enzyme aromatase cytochrome P-450^38^, and therefore the action at the tissue level might be driven by estradiol as well. In sex-stratified analyses, we found that serum estradiol and testosterone are associated with serum phosphate in both sexes. This is in line with a study in community-dwelling older men, where higher serum estradiol and testosterone concentrations were independently associated with lower serum phosphate concentrations, also after adjustment for FGF23 concentrations, one of the major phosphate regulating hormones^39,40^. Meng et al. also found an inverse association between sex-hormones and phosphate and could not attribute this to increased bone turnover since adjustments for bone mineral density and ALP did not influence the association^39^. Estradiol is able to induce phosphaturia in a PTH-independent pathway and may be a potent stimulus for FGF23 secretion^41^. Hence, the drop in estradiol associated with menopause could lead to reduced phosphate excretion by the kidneys, which may explain the higher serum phosphate concentrations after menopause as seen in our study as well as documented by others^5,18,20–23^. Most of the studies directly assessing the influence of sex steroids on serum phosphate concentrations focused on serum estradiol despite the increasing body of evidence that suggests a relationship between serum phosphate and testosterone^40,42^. As Meng et al. mentioned, there have not been many studies on the influence of testosterone on phosphate handling^42^. Investigating whether testosterone has potential direct effects on phosphate handling in the kidneys and intestines and on its regulatory hormones PTH, FGF23 and klotho will lead to a greater understanding of how testosterone influences the bone-kidney axis.

Since calcium and phosphate are mainly stored in bone, we assessed sex differences in calcium and phosphate concentrations in relation to bone turnover as reflected by serum ALP. However, adjustment for ALP concentrations, which were higher in women compared to men, as is known in the postmenopausal state due to estrogen deficiency^43^, did not influence our results. Although there was no availability of bone-specific ALP or other bone turnover markers, this observation suggests that a higher bone turnover in postmenopausal women compared to men does not fully explain the observed increases in serum calcium and phosphate concentrations.

Serum calcium and phosphate concentrations are associated with cardiometabolic diseases and mortality, and understanding the underlying mechanisms is a key priority. Lorenzo et al. have found serum calcium and the calcium-phosphate product but not phosphate to be associated with the incidence of type 2 diabetes in both sexes^44^. Moreover, Larsson et al. showed that a genetic predisposition to higher serum calcium concentrations was associated with increased myocardial infarction and coronary artery disease^45^. Dhingra et al. showed that serum phosphate concentrations, but not serum calcium concentrations, are associated with the composite endpoint of fatal and non-fatal cardiovascular disease (CVD) events in non-chronic kidney disease (CKD) subjects^22^ and a recent meta-analysis by Bai et al. in 120,269 subjects showed serum phosphate to be associated with all-cause mortality in men but not in women^46^. Lastly, in a large prospective study, Foley et al. found that higher serum phosphate concentrations in young adults are associated with higher scores of coronary artery calcium concentrations^47^. The higher serum concentrations of calcium and phosphate with the resulting higher calcium-phosphate product that we found in postmenopausal women compared to men of the same age may thus be clinically relevant. It may even be speculated that higher concentrations of serum calcium and phosphate after menopause may, in part, underlie the rise in CVD that is observed in women after menopause^48,49^.

The strength of this study is the availability of data in a large number of men and women in the Rotterdam Study. The large number of covariates, including serum estradiol, testosterone, vitamin D, ALP concentrations and use of hormone replacement therapy, allowed us to study the influence of these variables on calcium and phosphate homeostasis. Despite the relatively small sex differences in serum calcium and phosphate concentrations, our findings may improve our understanding of the underlying pathways for these sex differences. Moreover, our results may be relevant in light of the increasing number of studies showing that serum concentrations of calcium and phosphate in the general populations are related to multiple diseases and mortality^5,6,12,13^. Correcting for albumin in various ways in RS-I-1 has not influenced our findings of sex-based differences in total serum calcium concentrations.

This study has several limitations. We did not have availability of serum PTH and FGF23 concentrations and urinary excretion of calcium and phosphate. We did not study ionized calcium levels, which is the biologically active fraction of calcium and the fraction regulated by PTH^50^. Moreover, we had no measurements of sex hormone binding globulin, restricting us to analyses with total serum testosterone and estradiol concentrations and not with free sex hormone concentrations. Furthermore, the fact that serum phosphate in RS-I-1 in our study was determined in a non-fasting state may have influenced some of our findings^51^. There was no data on dietary intake of calcium and phosphate which could have influenced our results as well. Moreover, data on the duration of HRT use and current HRT use was not documented, which could have obscured a possible effect of HRT on serum calcium and phosphate concentrations. Lastly, the results are not generalizable to non-Caucasian populations and despite adjustment for multiple covariates, we cannot exclude the possibility of residual confounding.

To conclude, this study demonstrates sexual dimorphism in serum calcium and phosphate concentrations with postmenopausal women having significantly higher concentrations compared to men of similar age. Serum calcium and phosphate concentrations decline with age in both sexes. Serum estradiol and testosterone are inversely associated with serum phosphate in both sexes but sex differences seem to be explained in part by serum testosterone and not by estradiol. Serum estradiol and not serum testosterone is associated with serum calcium in both sexes and may play a role in the observed sex differences in serum calcium. Vitamin D is associated with serum calcium but does not appear to explain the observed sex differences. Based on the relations between serum calcium and phosphate concentrations and morbidity, especially cardiometabolic diseases and mortality, studies providing insight into mechanisms behind the origin of these sex differences may be of great relevance for public health and sex-based medicine.

## Supplementary Information

Supplementary Tables.

## Data Availability

Data can be obtained upon request. Requests should be directed towards the management team of the Rotterdam Study (datamanagement.ergo@erasmusmc.nl), which has a protocol for approving data requests. Because of restrictions based on privacy regulations and informed consent of the participants, data cannot be made freely available in a public repository.
