# Supplementary material for: Sexual dimorphisms in serum calcium and phosphate concentrations in the Rotterdam Study
Source: Sci Rep. 2023 May 23;13:8310. doi: 10.1038/s41598-023-34800-w (PMC10205794; doi:10.1038/s41598-023-34800-w)
Supplement: Supplementary file 1 — Supplementary Tables. [file 41598_2023_34800_MOESM1_ESM.docx]

**Sexual dimorphisms in serum calcium and phosphate concentrations in the Rotterdam Study**

Ariadne Bosman^1#^, W. Nadia H. Koek^1#^, Natalia Campos-Obando^1^, Bram C. J. van der Eerden^1^, M. Arfan Ikram^2^, André G. Uitterlinden^1,2^, Johannes P.T.M. van Leeuwen^1^, M. Carola Zillikens^1^*

*^1^ Erasmus MC, University Medical Center Rotterdam, Department of Internal Medicine, Rotterdam, the Netherlands*

*^2^ Erasmus MC, University Medical Center Rotterdam, Department of Epidemiology, Rotterdam, the Netherlands*

^#^ These authors contributed equally to this work

*Correspondence: Prof. dr. M. C. Zillikens. PO Box 2040 3000 CA Rotterdam, The Netherlands. E-mail address: m.c.zillikens@erasmusmc.nl +31-10-7040704

**Short title: S**exual dimorphism in serum calcium and phosphate

**Key words:** calcium; phosphate; ageing; sex differences.

**SUPPLEMENTARY**

**Supplementary Table 1. Baseline characteristics of RS-I-3 for men and women separately**

|  | Men | Women | P value |
| --- | --- | --- | --- |
| N | 1530 | 2093 |  |
| Age (years) | 71.8 (6.6) | 72.5 (7.0) | 0.005 |
| Calcium (mmol/L) | 2.41 (0.10) | 2.44 (0.10) | <0.001 |
| Phosphate (mmol/L) | 1.02 (0.14) | 1.17 (0.14) | <0.001 |
| Calcium-phosphate product (mmol^2^/L^2^) | 2.46 (0.38) | 2.86 (0.37) | <0.001 |
| 25(OH)D (nmol/L) | 56.0 (26.4) | 44.1 (23.2) | <0.001 |
| eGFR (mL/min/1.73 m^2^) | 72.2 (14.6) | 71.0 (13.8) | 0.01 |
| Estradiol (pmol/L) | 91.4 (35.9) | 33.3 (35.7) | <0.001 |
| Testosterone (nmol/L) | 17.47 (6.11) | 1.05 (0.92) | <0.001 |
| BMI (kg/m^2^) | 26.3 (3.2) | 27.3 (4.4) | <0.001 |
| Current smoking, n(%) | 276 (18.0%) | 302 (14.4%) | 0.003 |
| Ever HRT use, n(%) | - | 235 (15.1%) | - |
| Postmenopausal, n(%) | - | 2093 (100.0%) | - |

Continuous values are displayed as mean (standard deviation)), categorical variables are displayed in absolute counts (%). Differences between males and females were analyzed using an independent t test for continuous variables and χ2 test for categorical variables. Abbreviations: 25(OH)D, 25-hydroxyvitamin D; BMI, body mass index; eGFR, estimated glomerular filtration rate; HRT, hormone replacement therapy; RS, Rotterdam Study.

**Supplementary Table 2. Baseline characteristics of RS-II-1 for men and women separately**

|  | Men | Women | P value |
| --- | --- | --- | --- |
| N | 1078 | 1316 |  |
| Age (years) | 64.4 (7.5) | 64.8 (8.1) | 0.162 |
| Calcium (mmol/L) | 2.39 (0.09) | 2.42 (0.09) | <0.001 |
| Phosphate (mmol/L) | 1.00 (0.14) | 1.15 (0.14) | <0.001 |
| Calcium-phosphate product (mmol^2^/L^2^) | 2.39 (0.36) | 2.77 (0.36) | <0.001 |
| 25(OH)D (nmol/L) | 65.3 (28.8) | 58.8 (27.9) | <0.001 |
| eGFR (mL/min/1.73 m^2^) | 80.1 (15.1) | 79.0 (15.0) | 0.079 |
| Estradiol (pmol/L) | 130.2 (40.6) | 72.9 (63.8) | <0.001 |
| Testosterone (nmol/L) | 16.34 (5.79) | 0.87 (0.93) | <0.001 |
| BMI (kg/m^2^) | 26.9 (3.4) | 27.4 (4.4) | <0.001 |
| Current smoking, n(%) | 206 (19.1%) | 274 (20.8%) | 0.298 |
| Ever HRT use, n(%) |  | 264 (20.2%) |  |
| Postmenopausal, n(%) |  | 1294 (98.3%) |  |

Continuous values are displayed as mean (standard deviation)), categorical variables are displayed in absolute counts (%). Differences between males and females were analyzed using an independent t test for continuous variables and χ2 test for categorical variables. Abbreviations: 25(OH)D, 25-hydroxyvitamin D; BMI, body mass index; eGFR, estimated glomerular filtration rate; HRT, hormone replacement therapy; RS, Rotterdam Study.

**Supplementary Table 3. Baseline characteristics of RS-III-1 for men and women separately**

|  | Men | Women | P value |
| --- | --- | --- | --- |
| N | 1435 | 1806 |  |
| Age (years) | 56.8 (6.6) | 57.2 (7.0) | 0.080 |
| Calcium (mmol/L) | 2.45 (0.10) | 2.46 (0.11) | <0.001 |
| Phosphate (mmol/L) | 1.04 (0.15) | 1.19 (0.15) | <0.001 |
| Calcium-phosphate product (mmol^2^/L^2^) | 2.55 (0.41) | 2.93 (0.43) | <0.001 |
| 25(OH)D (nmol/L) | 60.0 (28.2) | 60.6 (27.7) | 0.540 |
| eGFR (mL/min/1.73 m^2^) | 88.1 (14.5) | 87.4 (14.2) | 0.168 |
| Estradiol (pmol/L) | 92.4 (36.8) | 85.7 (156.0) | <0.001 |
| Testosterone (nmol/L) | 14.48 (5.87) | 0.87 (0.52) | <0.001 |
| BMI (kg/m^2^) | 27.9 (4.0) | 27.7 (5.1) | 0.270 |
| Current smoking, n(%) | 323 (22.5%) | 416 (23.0%) | 0.629 |
| Ever HRT use, n(%) | - | 348 (19.6%) |  |
| Postmenopausal, n(%) | - | 1415 (78.3%) |  |

Continuous values are displayed as mean (standard deviation)), categorical variables are displayed in absolute counts (%). Differences between males and females were analyzed using an independent t test for continuous variables and χ2 test for categorical variables. Abbreviations: 25(OH)D, 25-hydroxyvitamin D; BMI, body mass index; eGFR, estimated glomerular filtration rate; HRT, hormone replacement therapy; RS, Rotterdam Study.

**Supplementary Table 4** Serum calcium and phosphate concentrations across consecutive decades stratified by sex in the pooled dataset of RS-I-3, RS-II-1 and RS-III-1

|  | Men | | | | | Women | | | | |
| --- | --- | --- | --- | --- | --- | --- | --- | --- | --- | --- |
| Decades  (yrs) | 45-54 | 55-64 | 65-74 | >75^*^ |  | 45-54 | 55-64 | 65-74 | >75^*^ |  |
| No. | 590 | 1708 | 1127 | 618 | p value | 701 | 2170 | 1351 | 993 | p value |
| Calcium (mmol/l) | 2.454 (2.445-2.462) | 2.419 (2.414-2.424) | 2.401 (2.395-2.406) | 2.392 (2.385-2.400) | 3.4x10^-7^ | 2.453 (2.445-2.461) | 2.447 (2.442-2.451) | 2.435 (2.430-2.440) | 2.438 (2.432-2.445) | 2.0x10^-6^ |
| Phosphate (mmol/l) | 1.060 (1.046-1.073) | 1.019 (1.012-1.026) | 1.016 (1.008-1.025) | 1.012 (1.005-1.026) | 0.002 | 1.175 (1.163-1.187) | 1.179 (1.173-1.185) | 1.170 (1.163-1.178) | 1.154 (1.145-1.163) | 1.9x10^-5^ |

Analyses were adjusted for BMI, eGFR, smoking and cohort. Values are depicted as mean with 95% CI. * Due to limited numbers of participants above 75 years this group ranges 75-96.7 years in men and 75-101 years in women. Abbreviations: BMI, body mass index; eGFR, estimated glomerular filtration rate; RS, Rotterdam Study.

**Supplementary Table 5. Influence of serum testosterone, estradiol and 25(OH)D on the association between age and serum calcium in men in the pooled dataset**

|  | Serum calcium (mmol/l) | |
| --- | --- | --- |
| N=4043 | Standardized Beta-coefficient age (95% CI) | % change beta-coefficient from model 1 |
| Model 1^a^ | -0.166 (-0.217 to -0.116) |  |
| Model 2^b^ | -0.153 (-0.203 to -0.102) | -7.8% |
| Model 3^c^ | -0.162 (-0.212 to -0.111) | -2.4% |
| Model 4^d^ | -0.138 (-0.188 to -0.087) | -16.9% |
| Model 5^e^ | -0.159 (-0.210 to -0.109) | -4.2% |

βs were obtained from linear regression models and expressed per 1-SD increase in serum estradiol, testosterone or 25(OH)D. ^a^Model 1: adjusted for age in decades, cohort, BMI, eGFR and smoking. ^b^Model 2: model 1 and estradiol. ^c^Model 3: model 1 and testosterone. ^d^Model 4: model 1 and estradiol and testosterone. ^e^Model 5: model 1 and 25(OH)D. Abbreviations: BMI, body mass index; eGFR, estimated glomerular filtration rate; RS, Rotterdam Study.

**Supplementary Table 6. Baseline characteristics of women in the pooled dataset, stratified by menopausal status**

|  | Premenopausal | Postmenopausal | P value |
| --- | --- | --- | --- |
| N | 413 | 4802 |  |
| Age (years) | 51.2 (3.2) | 66.5 (9.3) | <0.001 |
| Calcium (mmol/L) | 2.44 (0.12) | 2.44 (0.10) | 0.879 |
| Phosphate (mmol/L) | 1.14 (0.16) | 1.17 (0.14) | <0.001 |
| Calcium-phosphate product (mmol^2^/L^2^) | 2.80 (0.45) | 2.87 (0.39) | 0.004 |
| 25(OH)D (nmol/L) | 61.5 (28.9) | 52.9 (26.9) | <0.001 |
| eGFR (mL/min/1.73 m^2^) | 93.6 (11.6) | 77.4 (15.6) | <0.001 |
| Estradiol (pmol/L) | 221.7 (258.3) | 47.7 (57.4) | <0.001 |
| Testosterone (nmol/L) | 0.85 (0.38) | 0.95 (0.84) | <0.001 |
| BMI (kg/m^2^) | 27.4 (5.3) | 27.5 (4.6) | 0.956 |
| Current smoking, n(%) | 81 (19.6%) | 911 (19.0%) | 0.003 |
| Ever HRT use, n(%) | - | 847 (18.2%) |  |

Continuous values are displayed as mean (standard deviation)), categorical variables are displayed in absolute counts (%). Differences between premenopausal and postmenopausal women were analyzed using an independent t test for continuous variables and χ2 test for categorical variables. Abbreviations: 25(OH)D, 25-hydroxyvitamin D; BMI, body mass index; eGFR, estimated glomerular filtration rate; HRT, hormone replacement therapy .

**Supplementary Table 7. Baseline characteristics of RS-I-1 for men and women separately**

|  | Men | Women | P value |
| --- | --- | --- | --- |
| N | 1009 | 1679 |  |
| Age (years) | 69.6 (8.0) | 71.2 (9.0) | <0.001 |
| Calcium (mmol/L) | 2.36 (0.14) | 2.37 (0.13) | 0.002 |
| Corrected calcium (mmol/L) | 2.35 (0.13) | 2.37 (0.13) | <0.001 |
| Serum phosphate (mmol/L) | 1.09 (0.19) | 1.23 (0.17) | <0.001 |
| Calcium-phosphate product (mmol^2^/L^2^) | 2.57 (0.50) | 2.92 (0.47) | <0.001 |
| Alkaline Phosphatase IU/L | 80.4 (28.2) | 82.9 (26.8) | <0.001 |
| Albumin (g/L) | 42.2 (2.6) | 42.1 (2.5) | 0.169 |
| 25(OH)D (nmol/L)† | 69.1 (27.3) | 59.8 (25.5) | <0.001 |
| 1,25(OH)_2_D_3_(pmol/L) † | 109.1 (29.9) | 105.7 (28.9) | 0.069 |
| eGFR (mL/min/1.73 m^2^) | 79.8 (15.7) | 69.4 (14.6) | <0.001 |
| Estradiol (pmol/L)‡ | 48.4 (22.9) | 21.1 (14.6) | <0.001 |
| Testosterone (nmol/L)‡ | 10.7 (3.3) | 1.4 (0.7) | <0.001 |
| BMI (kg/m^2^) | 25.7 (3.0) | 26.6 (4.2) | <0.001 |
| Current smoking, n(%) | 322 (31.9%) | 309 (18.4%) | <0.001 |
| Ever HRT use, n(%) | - | 252 (15.5%) |  |
| Postmenopausal, n(%) | - | 1679 (100%) |  |

Continuous values are displayed as mean (standard deviation)), categorical variables are displayed in absolute counts (%). Differences between males and females were analyzed using an independent t test for continuous variables and χ2 test for categorical variables. Abbreviations: 25(OH)D, 25-hydroxyvitamin D; 1,25(OH)_2_D_3_, 1,25-dihydroxyvitamin D; BMI, body mass index; eGFR, estimated glomerular filtration rate; HRT, hormone replacement therapy; RS, Rotterdam Study.

**†** 25(OH)D and 1,25(OH)_2_D_3_ concentrations available in 426 men and 620 women from RS-I-1

‡ Estradiol and testosterone concentrations available in 241 men and 228 women from RS-I-1

**Supplementary table 8. Association between sex and age, and serum calcium and phosphate levels in the RS-I-1**

|  |  | Serum calcium (mmol/L) | | Serum phosphate (mmol/L) | |
| --- | --- | --- | --- | --- | --- |
| N=2688 |  | Standardized Beta-coefficient (95% CI) | P value | Standardized Beta-coefficient (95% CI) | P value |
|  | Sex | 0.175 (0.094 to 0.257) | <0.001 | 0.800 (0.723 to 0.877) | <0.001 |
|  | Age | -0.033 (-0.078 to 0.012) | 0.145 | -0.050 (-0.093 to -0.008) | 0.020 |

βs were obtained from linear regression models. All models were adjusted for sex, age in decades, BMI, eGFR and smoking. Abbreviations: BMI, body mass index; eGFR, estimated glomerular filtration rate; RS, Rotterdam Study.

**Supplementary table 9. Influence of serum 25(OH)D, 1.25(OH)_2_D and ALP on sex differences in serum calcium and phosphate concentrations in RS-I-1**

|  |  | Serum calcium (mmol/l) | | Serum phosphate (mmol/l) | |
| --- | --- | --- | --- | --- | --- |
| N=1046* |  | Standardized Beta-coefficient sex (95% CI) | % change  beta-coefficient from model 1 | Standardized Beta-coefficient sex (95% CI) | % change  beta-coefficient from model 1 |
|  | Model 1^a^ | 0.234 (0.103 to 0.364) |  | 0.865 (0.743 to 0.986) |  |
|  | Model 2^b^ | 0.215 (0.082 to 0.349) | -8.1% | 0.841 (0.716 to 0.966) | -2.8% |
|  | Model 3^c^ | 0.235 (0.104 to 0.366) | +0.4% | 0.864 (0.742 to 0.986) | -0.1% |
|  | Model 4^d^ | 0.207 (0.073 to 0.41) | -11.5% | 0.842 (0.718 to 0.967) | -2.7% |
|  | Model 5^e^ | 0.227 (0.096 to 0.358) | -3.0% | 0.866 (0.743 to 0.988) | +0.1% |

βs were obtained from linear regression models and expressed per 1-SD increase in serum 25(OH)D, 1.25(OH)_2_D_3_, or ALP. *25(OH)D and 1,25(OH)_2_D_3_ concentrations were available in 426 men and 620 women from RS-I-1 ^a^Model 1: adjusted for age in decades, BMI, eGFR and smoking. ^b^Model 2: model 1 and 25(OH)D. ^c^Model 3: model 1 and 1.25(OH)_2_D_3_. ^e^Model 4: model 1 and 25(OH)D and 1.25(OH)_2_D_3_. ^e^Model 5: model 1 and ALP. Abbreviations: 25(OH)D, 25-hydroxyvitamin D; 1,25(OH)_2_D_3_, 1,25-dihydroxyvitamin D; BMI, body mass index; eGFR, estimated glomerular filtration rate; RS, Rotterdam Study

**Supplementary table 10. Beta-coefficients for serum calcium and phosphate levels, according to covariates, in men and women separately in RS-I-1**

|  |  | Serum calcium (mmol/L) | | Serum phosphate (mmol/L) | |
| --- | --- | --- | --- | --- | --- |
| Men (n=1009) |  | Standardized Beta-coefficient (95% CI) | P value | Standardized Beta-coefficient (95% CI) | P value |
|  | Age (per decade) | -0.030 (-0.110 to 0.050) | 0.462 | 0.008 (-0.073 to 0.088) | 0.850 |
|  | 25(OH)D***** | -0.031 (-0.124 to 0.062) | 0.519 | -0.072 (-0.170 to 0.027) | 0.155 |
|  | 1,25(OH)_2_D***** | 0.087 (-0.008 to 0.181) | 0.072 | -0.025 (-0.126 to 0.075) | 0.620 |
|  | ALP | -0.047 (-0.109 to 0.014) | 0.132 | -0.025 (-0.087 to 0.036) | 0.422 |
|  |  |  |  |  |  |
| Women (n=1629) |  | Standardized Beta-coefficient (95% CI) | P value | Standardized Beta-coefficient (95% CI) | P value |
|  | Age (per decade) | -0.019 (-0.076 to 0.038) | 0.512 | -0.088 (-0.147 to -0.030) | 0.003 |
|  | 25(OH)D**†*** | -0.026 (-0.104 to 0.051) | 0.506 | -0.039 (-0.121 to 0.043) | 0.355 |
|  | 1,25(OH)_2_D***** | 0.036 (-0.037 to 0.109) | 0.333 | -0.026 (-0.103 to 0.051) | 0.514 |
|  | ALP | 0.040 (-0.009 to 0.089) | 0.111 | 0.007 (-0.044 to 0.057) | 0.800 |

βs were obtained from linear regression models and expressed per 1-SD increase 25(OH)D, 1,25(OH)_2_D_3_ and alkaline phosphatase. All models were adjusted for age, BMI, eGFR and smoking (and previous HRT use in women). Abbreviations: 25(OH)D, 25-hydroxyvitamin D; 1,25(OH)_2_D_3_, 1,25-dihydroxyvitamin D; BMI, body mass index; eGFR, estimated glomerular filtration rate; HRT, hormone replacement therapy; RS, Rotterdam Study.

*****25(OH)D and 1,25(OH)_2_D_3_ concentrations were available in 426 men and 620 women from RS-I-1
